# Residual malaria in Jazan region, southwestern Saudi Arabia: the situation, challenges and climatic drivers of autochthonous malaria

**DOI:** 10.1186/s12936-021-03846-4

**Published:** 2021-07-13

**Authors:** Hesham M. Al-Mekhlafi, Aymen M. Madkhali, Khalid Y. Ghailan, Ahmed A. Abdulhaq, Ahmad Hassn Ghzwani, Khalid Ammash Zain, Wahib M. Atroosh, Alkhansa Alshabi, Hussein A. Khadashi, Majid A. Darraj, Zaki M. Eisa

**Affiliations:** 1 Medical Research Centre, Jazan University, Jazan, Kingdom of Saudi Arabia; 2 Department of Parasitology, Faculty of Medicine, University of Malaya, 50603 Kuala Lumpur, Malaysia; 3 Department of Parasitology, Faculty of Medicine and Health Sciences, Sana’a University, Sana’a, Yemen; 4 Department of Medical Laboratory Technology, Faculty of Applied Medical Sciences, Jazan University, Jazan, Kingdom of Saudi Arabia; 5 Faculty of Public Health and Tropical Medicine, Jazan University, Jazan, Kingdom of Saudi Arabia; 6 Department of Microbiology and Parasitology, Faculty of Medicine and Health Sciences, University of Aden, Aden, Yemen; 7 Department of Internal Medicine, Faculty of Medicine, Jazan University, Jazan, Kingdom of Saudi Arabia; 8 Saudi Centre for Disease Prevention and Control, Ministry of Health, Jazan, Kingdom of Saudi Arabia

**Keywords:** Malaria, Climatic factors, Elimination, Infectious diseases, Jazan, Saudi Arabia

## Abstract

**Background:**

Saudi Arabia and Yemen are the only two countries in the Arabian Peninsula that are yet to achieve malaria elimination. Over the past two decades, the malaria control programme in Saudi Arabia has successfully reduced the annual number of malaria cases, with the lowest incidence rate across the country reported in 2014. This study aims to investigate the distribution of residual malaria in Jazan region and to identify potential climatic drivers of autochthonous malaria cases in the region.

**Methods:**

A cross-sectional study was carried out from 1 April 2018 to 31 January 2019 in Jazan region, southwestern Saudi Arabia, which targeted febrile individuals attending hospitals and primary healthcare centres. Participants’ demographic data were collected, including age, gender, nationality, and residence. Moreover, association of climatic variables with the monthly autochthonous malaria cases reported during the period of 2010–2017 was retrospectively analysed.

**Results:**

A total of 1124 febrile subjects were found to be positive for malaria during the study period. Among them, 94.3 and 5.7% were infected with *Plasmodium falciparum* and *Plasmodium vivax*, respectively. In general, subjects aged 18–30 years and those aged over 50 years had the highest (42.7%) and lowest (5.9%) percentages of malaria cases. Similarly, the percentage of malaria-positive cases was higher among males than females (86.2 vs 13.8%), among non-Saudi compared to Saudi subjects (70.6 vs 29.4%), and among patients residing in rural rather than in urban areas (89.8 vs 10.2%). A total of 407 autochthonous malaria cases were reported in Jazan region between 2010 and 2017. Results of zero-inflated negative binomial regression analysis showed that monthly average temperature and relative humidity were the significant climatic determinants of autochthonous malaria in the region.

**Conclusion:**

Malaria remains a public health problem in most governorates of Jazan region. The identification and monitoring of malaria transmission hotspots and predictors would enable control efforts to be intensified and focused on specific areas and therefore expedite the elimination of residual malaria from the whole region.

**Supplementary Information:**

The online version contains supplementary material available at 10.1186/s12936-021-03846-4.

## Background

Malaria, one of the most common life-threatening infectious diseases, is still a major public health problem worldwide, particularly in tropical and sub-tropical regions. According to the World Health Organization (WHO), about 229 million new cases of malaria were reported worldwide in 2019 and over 3.4 billion people are at risk of infection [1]. Almost 94% of the malaria cases were reported in the WHO African Region (AFR), while 3.0 and 2.2% of the cases were recorded in the WHO Southeast Asia Region (SEAR) and Eastern Mediterranean Region (EMR), respectively [1]. Moreover, approximately 409,000 malaria deaths occurred worldwide in 2019, with sub-Saharan Africa accounting for about 95% of all global malaria deaths [1, 2]. *Plasmodium falciparum* is considered the most virulent and prevalent *Plasmodium* species, accounting for 99.7, 69 and 62.8% of the reported malaria cases in the AFR, EMR and SEAR regions, respectively [3]. Furthermore, it has been estimated that about 14.3 million malaria cases in 2018 were attributable to *Plasmodium vivax* and that 3.3 billion people are at risk of vivax malaria infection worldwide [4].

In Saudi Arabia, the national malaria control programme, which was established in 1948, has achieved a tremendous reduction in the annual number of malaria cases, and malaria is now restricted to the southwestern parts of the country, which includes the Aseer and Jazan regions. The number of autochthonous/indigenous (locally transmitted) malaria cases in Saudi Arabia decreased dramatically between 2000 and 2014, from 511 in 2000 to just 30 in 2014, and the country has been included in the E-2020 WHO initiative, which is focused on achieving a target of zero autochthonous cases by 2020 [5]. However, malaria cases increased after 2014, with 5,382 malaria cases reported in 2016, including 272 locally transmitted cases (270 falciparum and two vivax malaria) [6, 7]. In the global context, this number of cases is considered high and the country therefore remains determined to make vigorous efforts to achieve E-2020 status.

Progress toward malaria elimination in Saudi Arabia until 2014 has been investigated by several researchers [8, 9]. However, there is a scarcity of information about the frequency and distribution of malaria cases in Jazan region after 2014. Therefore, this study aims to fill this gap in knowledge and to elucidate the situation regarding the level of residual malaria endemicity in the region, as well as to investigate associations of autochthonous malaria with selected climatic (meteorological) factors over a period of 8 years (2010–2017). In malaria elimination settings, such information is crucial to identify the challenges and further research needs towards the elimination of malaria in the targeted areas.

## Methods

### Study design

In the first part of this study, a health facility-based survey was carried out between April 2018 and January 2019. Malaria cases were detected through passive case detection among symptomatic febrile patients who visited government hospitals in different governorates of Jazan region. In the second part, association between monthly autochthonous malaria cases and climatic variables was assessed retrospectively for the period 2010 to 2017.

### Study area and study population

The Kingdom of Saudi Arabia lies in the furthermost part of southwestern Asia, bordered by the Red Sea to the west, the Arabian Gulf, United Arab Emirates, and Qatar to the east, Yemen and Oman to the south, and Kuwait, Iraq and Jordan to the north. It is the largest country in the Arab Peninsula, occupying about four-fifths of the Peninsula with a total surface area of approximately 2,000,000 km^2^ and a total population of about 34 million [10]. Geographically, Saudi Arabia can be divided into two distinct zones: the rain-fed highlands of the western and southwestern regions (Sarawat Mountains) and the vast arid and extra-arid lands of the interior (Najd) [11]. Although Saudi Arabia largely has a desert climate, the climatic conditions differ from one region to another due to the diverse topography of the Kingdom. Generally, the interior parts of the country, which are mostly desert, experience dry hot summers and cold winters, while the coastal areas have high temperature and high humidity and the southwestern part of the country has a moderate climate [12].

**Fig. 1:**
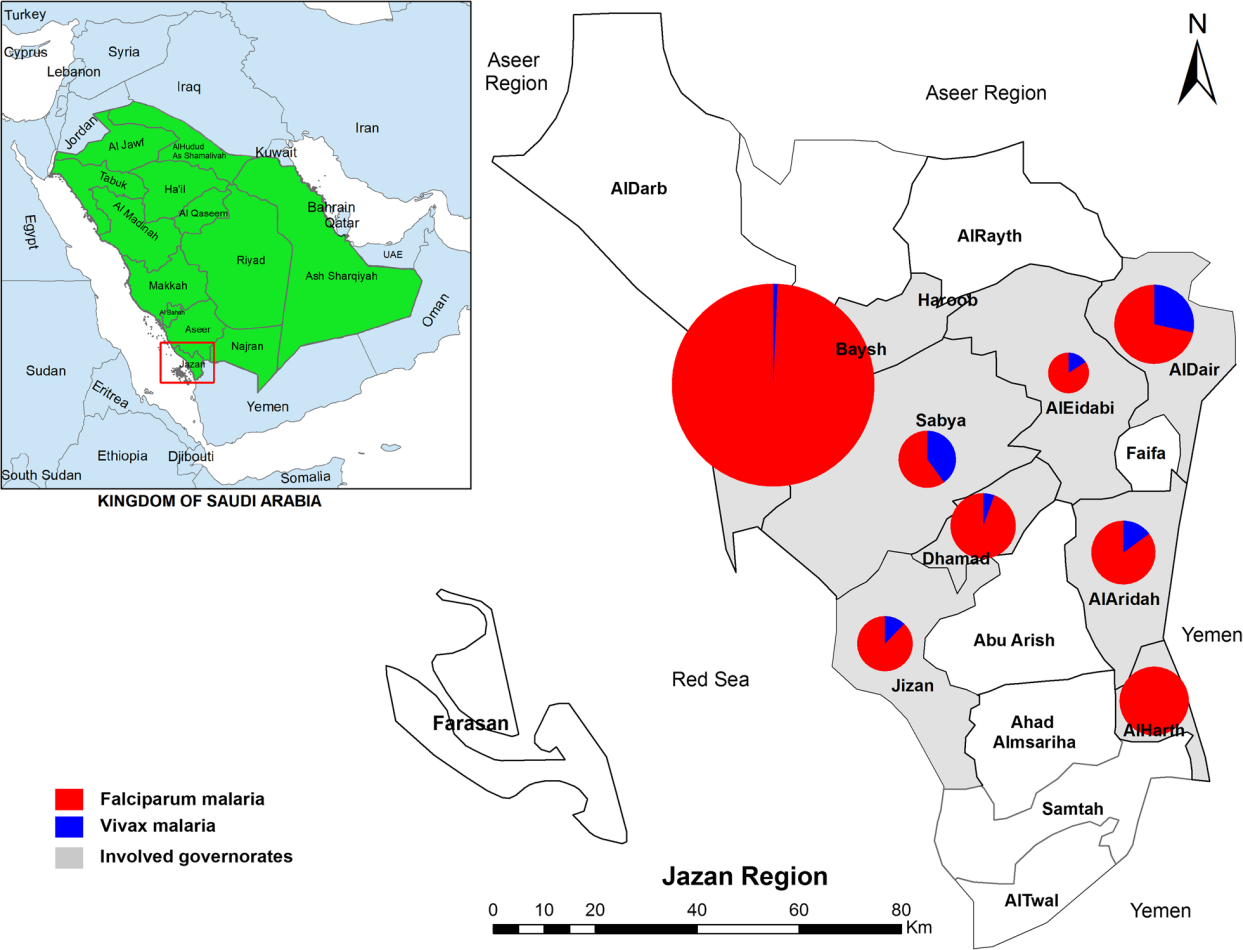
A geographic map showing the governorates involved in the study (8 governorates) in Jazan region, southwestern Saudi Arabia. Pie size is proportional to malaria frequency at each governorate

Jazan region is located in the southwest corner of the Kingdom of Saudi Arabia between longitude 42.7076° E and latitude 17.4751° N. Jazan region is considered the smallest region in the Kingdom, but it has the highest population density in the country, occupying a total land area of approximately 11,671 km^2^ and having a total population of about 1.8 million. The region consists of three distinct zones: a highland zone (Fayfa Mountains, a part of the Sarawat range) at an elevation of 2500–3000 m above sea level and with rainfall of more than 200 mm/year; a hill zone at an elevation of 400–600 m above sea level and with rainfall of less than 200 mm/year; and, a coastal plain (stretching 300 km along the southern Red Sea coast and also including more than 100 islands) at an altitude of less than 400 m above sea level and with little of sporadic rain [13, 14]. The region contains many valleys (Wadi), such as Wadi Lajab and Wadi Baysh. In addition, there are a few streams in the region as well as 15 dams for the provision of drinking water to cities in the surrounding areas and irrigation water for agricultural activities [15]. Jazan region is divided into 17 governorates, and eight of them were included in this study (Fig. 1).

With regards to the malaria profile of the country, Saudi Arabia can be classified generally into four topographical categories as follows: (1) centre of the country (non-malarious areas); (2) northern and eastern regions (no malaria transmission, but *Anopheles superpictus* and *Anopheles stephensi* are still present as possible vectors); (3) western region (sporadic malaria occurrence); and, (4) southern and southwestern regions (low malaria incidence rates) [7, 8]. The highest number of malaria cases in Saudi Arabia occurred in 1998, when 36,159 cases were reported of which 61% were detected in Jazan [16]. Jazan region is currently the only region with indigenous malaria cases with few foci for malaria transmission [9, 17]. *Anopheles arabiensis* is the main vector for malaria in the region; however, other species, which are known to be competent malaria vectors, have also been recorded, including *Anopheles sergentii*, *Anopheles dthali*, *Anopheles fluviatilis*, *An. stephensi*, *An*. *superpictus*, and *Anopheles culicifacies* [18, 19]. Although mosquitoes can be found throughout the year, the presence of *Anopheles* usually peaks following the rainy season that falls during October to December [20, 21].

Saudi Arabia adopted artemisinin-based combination therapy (ACT) as its anti-malarial drug policy in 2007 [7]. ACT plus primaquine is used for the treatment of uncomplicated falciparum malaria, with artesunate plus sulfadoxine–pyrimethamine (AS + SP) and a combination of artemether–lumefanthrine (AL) as a first-line and second-line treatment, respectively. For severe falciparum malaria, parenteral artesunate and artemether are used as a first-line and second-line treatment, respectively [7, 22]. According to the policy, parenteral quinine therapy is the preferred option in cases of ACT therapeutic failure or when ACT is not available or contra-indicated in the first trimester of pregnancy [7]. On the other hand, vivax malaria is treated with a combination of chloroquine and primaquine for providing radical cure [7, 23], whereas chloroquine-resistant vivax malaria is treated with ACT combined with primaquine [7].

From 1 April 2018 to 31 January 2019, blood samples were collected from febrile patients presenting for healthcare at participating healthcare facilities in Jazan region. All patients who were found positive for malaria were invited to take part in the study regardless of their gender, age and nationality. Demographic data about the participants including age, gender, nationality, and residential address (village or town and governorate) were collected by using a standard semi-structured questionnaire or from patients’ medical records. The data were extracted from the patients’ medical records by trained research assistants on a weekly basis and entered into standard Excel spreadsheets prepared for the study. Laboratory diagnosis data including malaria status, and *Plasmodium* species and parasite density for malaria-positive patients, as well as the date of blood examination were also extracted. Moreover, whole blood samples and Giemsa-stained blood films of all malaria-positive subjects examined during the study period and who gave their consent to participate were collected from the respective healthcare facilities for further examination. In addition, blood samples of randomly selected malaria-negative subjects were also collected and re-examined.

### Blood sampling and examination

About 2–3 ml of venous blood was collected from each participant into an EDTA tube clearly labelled with the patient’s unique reference number, name, age and gender. Next, a drop of blood was subjected to a rapid diagnostic test (RDT) using the AMP Rapid Test-Malaria p.f./pan kit (AMEDA Labordiagnostik GmbH, Austria). Then, thin and thick blood films were prepared and stained with 3% of buffer-diluted Giemsa stain for 45 min, and then examined by light microscopy for the presence of malaria parasites. Infection status and parasite density were assessed using the thick films while parasite species and stages were assessed on thin films. A thick blood film was considered negative if no malaria parasites were detected after examining 200 high power fields under a 100 × objective [24].

Parasite density (parasitaemia), expressed as the number of parasites per µl of blood, was determined by counting the asexual stages against 200 white blood cells (WBCs) on the thick blood film, and then multiplying by 40, based on the WHO-recommended average of 8,000 WBCs per μl of blood in every human being [24]. The level of parasite density (parasitaemia) was graded as low (< 100 parasites/μl of blood), moderate (100–9999 parasites/μl of blood), and severe (≥ 10,000 parasites/μl of blood). Archived malaria positive slides were also collected from respective health centres and re-examined; parasite species and parasitaemia were recorded. For quality control, 25% of all slides were randomly selected and re-examined by a second reader blinded to the first results. The final results were calculated on the basis of the average of the two readings. Where results were discordant (25% in parasitaemia), a final decision was reached by a third expert microscopist.

### Data management and analysis

Data were double entered by two different research assistants into Microsoft Office Excel 2010 spreadsheets. Then, a third researcher crosschecked the two datasets for accuracy and created a single dataset for analysis. Climatic data for Jazan region, including mean minimum, maximum and average temperature, mean relative humidity, mean wind speed, mean atmospheric pressure, and monthly aggregate rainfall, sandstorm and dust haze events were provided by Sabya governorate’s weather station located in central Jazan, and from the annual statistical documents published by the Ministry of Environment, Water, and Agriculture – Saudi Arabia (https://mewa.gov.sa/ar/Pages/default.aspx) [15, 25], and the General Authority for Statistics (https://www.stats.gov.sa/en/258) [10].

Data analysis was performed using IBM SPSS Statistics version 27.0—Essentials for R (IBM Corporation, NY, USA). The demographic characteristics of the participants were treated as categorical variables and presented as frequencies and percentages, while the quantitative variables were presented either as mean (standard deviation, SD) or median (interquartile range, IQR). Pearson’s Chi-square test was used for 2 × 1 contingency table analysis to test the associations between malaria infection and demographic characteristics including age, gender, nationality, and residence [26].

For each climatic variable, a monthly average or aggregate was calculated. Malaria cases were grouped by month of onset. In Jazan region, malaria is mostly imported, however, only locally transmitted cases reported between 2010 and 2017 were considered in this study in order to justify potential relationship with local climatic variables. Pearson’s correlation coefficients were calculated to determine the correlation between malaria cases and the climatic variables. For biological reasons such as the development and survival of mosquitoes and the development of the parasite within the mosquito, different lags (0, 1, 2, 3 and 4) were considered for climatic variables to determine the maximum significant correlations. Since malaria cases was a countable variable with excessive zero counts, and the cases were over-dispersed with a variance greater than the mean, zero-inflated negative binomial regression (ZINB) was applied to identify the relationship between malaria cases and climatic factors at different monthly lags [27].

The covariates were selected based on multicollinearity (r > 0.80) and the strength of their statistical correlation with malaria cases (Additional file 1: Table S1). For each variable, the lag that showed the highest significant correlation with malaria cases was included in the ZINB analysis. Moreover, due to the multicollinearity problem detected between minimum temperature and relative humidity, two separate models were generated. Model A included minimum temperature but no relative humidity, and Model B for the converse. Moreover, both models included maximum temperature at lag 1, average temperature at lag 0, atmospheric pressure at lag 1, wind speed at lag 1, number of sandstorms at lag 0, and number of dust haze events at lag 2 as independent variables. Additional models that included lags with the second highest significant correlation for some variables (e.g. temperature indices, relative humidity and wind speed) were also considered in model fitting. The Akaike’s information criterion (AIC) was used to compare goodness-of-fit between models. All variables that reached a *P* value of < 0.10 in the best ZINB models favored by AIC were included in the final reduced model (Model C). To quantify the effects of the included climatic variables, influences or percentage change (e^β^ − 1) were computed [28]. A *P* value of < 0.05 was considered statistically significant for all statistical tests.

## Results

### Frequency and distribution of malaria cases

**Fig. 2:**
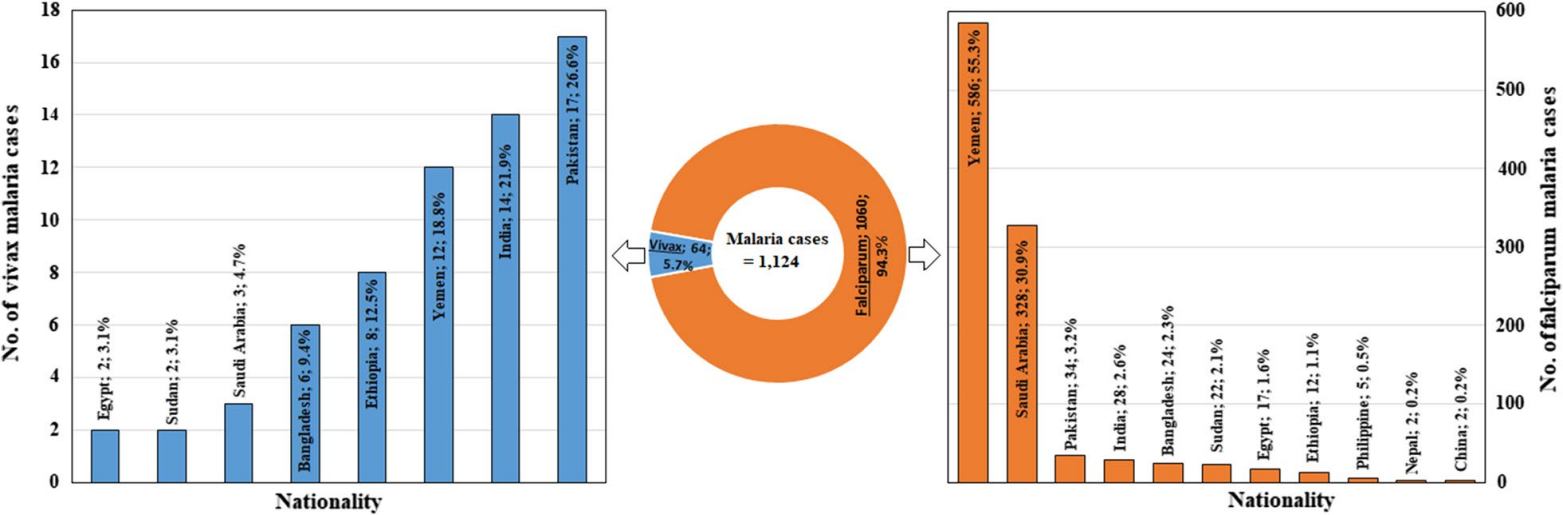
Distribution of falciparum and vivax malaria cases reported among febrile patients in Jazan region according to patients’ nationality

A total of 1124 febrile subjects were found to be positive for malaria: 1060 (94.3%) were infected with *P. falciparum* and 5.7% (64/1124) had *P. vivax* (Fig. 2). With regards to parasitaemia, 27.1, 26.7 and 46.2% of the patients with falciparum malaria had severe, moderate, and low parasitaemia, respectively. The median (IQR) parasitaemia of falciparum malaria was 1123 (456, 10,374) parasites/μl of blood. The asexual *P. falciparum* parasitaemia from the samples collected ranged from 113 to 33,472 parasites/μl of blood, with a geometric mean of 5721 parasites/μl. On the other hand, 25.0, 37.5 and 37.5% of the patients with vivax malaria had severe, moderate and low parasitaemia, respectively. The median (IQR) parasitaemia of vivax malaria was 2084 (564, 9,076) parasites/μl of blood.

Figure 1 shows the distribution of malaria according to residence. Overall, 6.4% of the confirmed cases were in Baysh followed by Aldair governorate (6.1%), while only 1.0% were in Alaridah governorate. Moreover, the majority (45.3%; 29/64) of vivax malaria cases were in Aldair followed by Sabya (18.8%; 12/64) governorate, while no cases were reported in Alharth governorate. Figure 2 displays the distribution of malaria cases according to nationality. Over half (54.2%; 609/1124) of the cases were among Yemeni subjects followed by Saudis (29.4%), while the lowest number of cases was found to be among Chinese and Nepalese patients (two cases each). Interestingly, about one quarter (26.6%; 17/64) of the vivax malaria cases were among Pakistanis followed by Indians (21.9%; 14 cases), and Yemenis (18.8%; 12 cases).

**Fig. 3:**
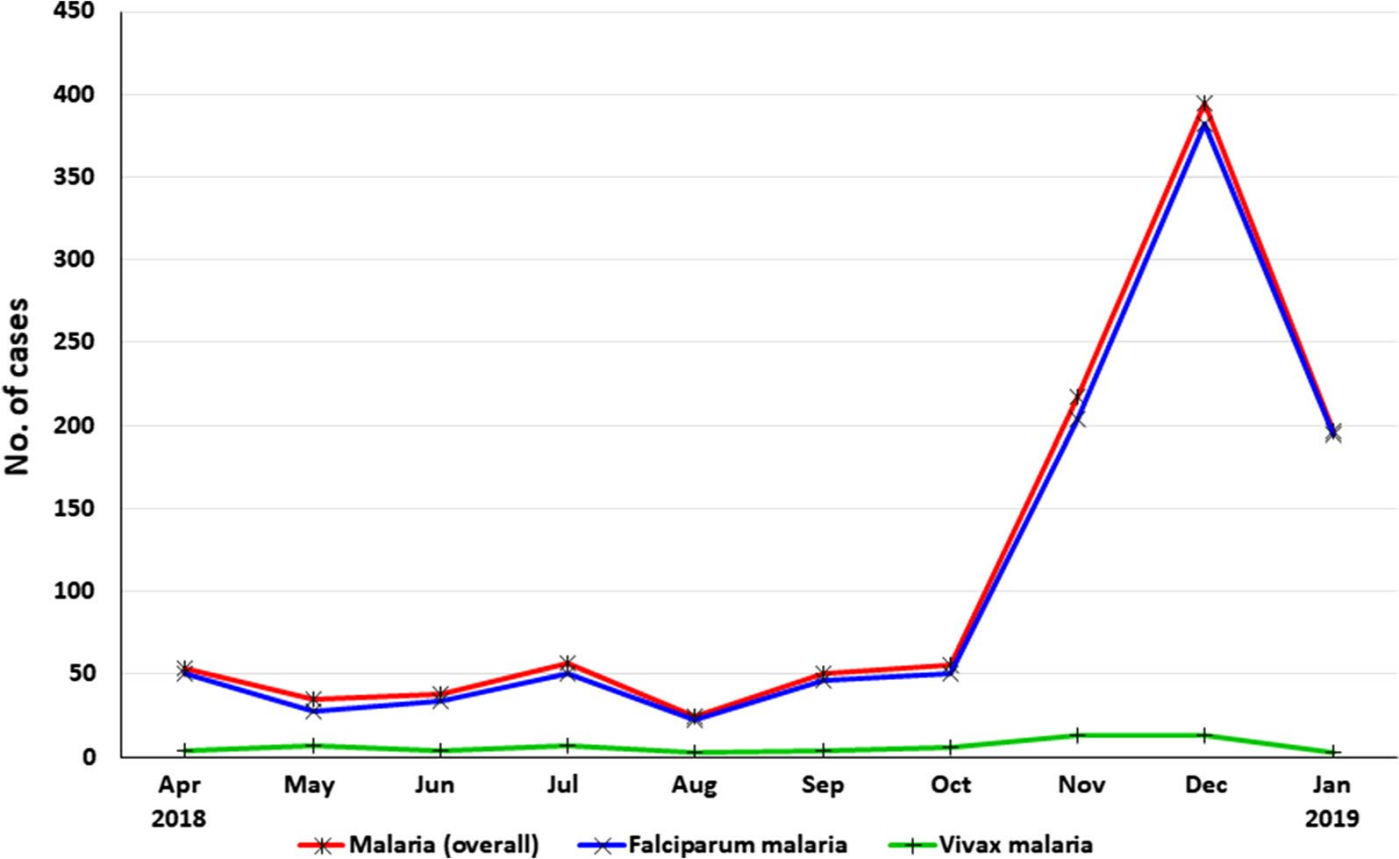
Monthly distribution of malaria cases among febrile patients in Jazan region during the study period

Figure 3 shows that the monthly distribution of malaria cases during the study period. The results showed continuous transmission of malaria throughout the year. Due to an outbreak in Baysh governorate, the percentage of malaria cases increased from 56 in October 2018 to 395 in December 2018. Nonetheless, the monthly malaria cases reported by this study corresponds to the known seasonal transmission of malaria in the region.

Table 1 shows the distribution of malaria cases according to demographic characteristics of the study subjects. The results showed that the percentage of confirmed cases was significantly higher among the males (86.2%) compared to females (13.8%) (*P* < 0.001). Also, subjects aged 18–30 years had the highest percentage (42.7%) of confirmed malaria cases followed by those aged 31–40 years (23.8%) while those aged over 50 years had the lowest (5.9%) when compared with other age groups. With regards to nationality, 70.6% of confirmed malaria cases were non-Saudis and 29.4% were Saudi subjects and the difference was statistically significant (*P* < 0.001). Similarly, a significantly higher percentage of malaria cases was found among subjects from rural areas (89.8%) compared to those from urban areas (10.2%). With regards to species, vivax malaria cases were not found among young children (aged below 10 years) and older persons (aged ≥ 65 years). Interestingly, four cases (6.3%) only of vivax malaria were among female patients, while the rest of the cases (60 cases; 93.7%) were found among their male counterparts. As regards nationality, three cases (4.7%) only of vivax malaria were among Saudis, while 61 cases (95.3%) were found among non-Saudis. More than three-quarters (78.1%; 50/64) of the vivax malaria cases were among rural subjects compared to 21.9% among their urban counterparts.

**Table 1:** Distribution of malaria confirmed cases according to demographic factors of febrile patients in Jazan region (n = 1124)

Variables	Falciparum malaria (n = 1060)	Vivax malaria (n = 64)	Overall malaria
Age groups (years)			
< 18 (children)	146 (13.8)	3 (4.7)	149 (13.3)
18–30	458 (43.2)	22 (34.4)	480 (42.7)
31–40	247 (23.3)	21 (32.8)	268 (23.8)
41–50	145 (13.7)	16 (25.0)	161 (14.3)
> 50	64 (6.0)	2 (3.1)	66 (5.9)
Gender			
Female	151 (14.2)	4 (6.3)	155 (13.8)
Male	909 (85.8)	60 (93.8)	969 (86.2)
Residence			
Urban	101 (9.5)	14 (21.9)	115 (10.2)
Rural	959 (90.5)	50 (78.1)	1009 (89.8)
Nationality			
Saudis	328 (30.9)	3 (4.7)	331 (29.4)
Non-Saudis	732 (69.1)	61 (95.3)	793 (70.6)

### Correlation of autochthonous malaria with climate parameters

**Fig. 4:**
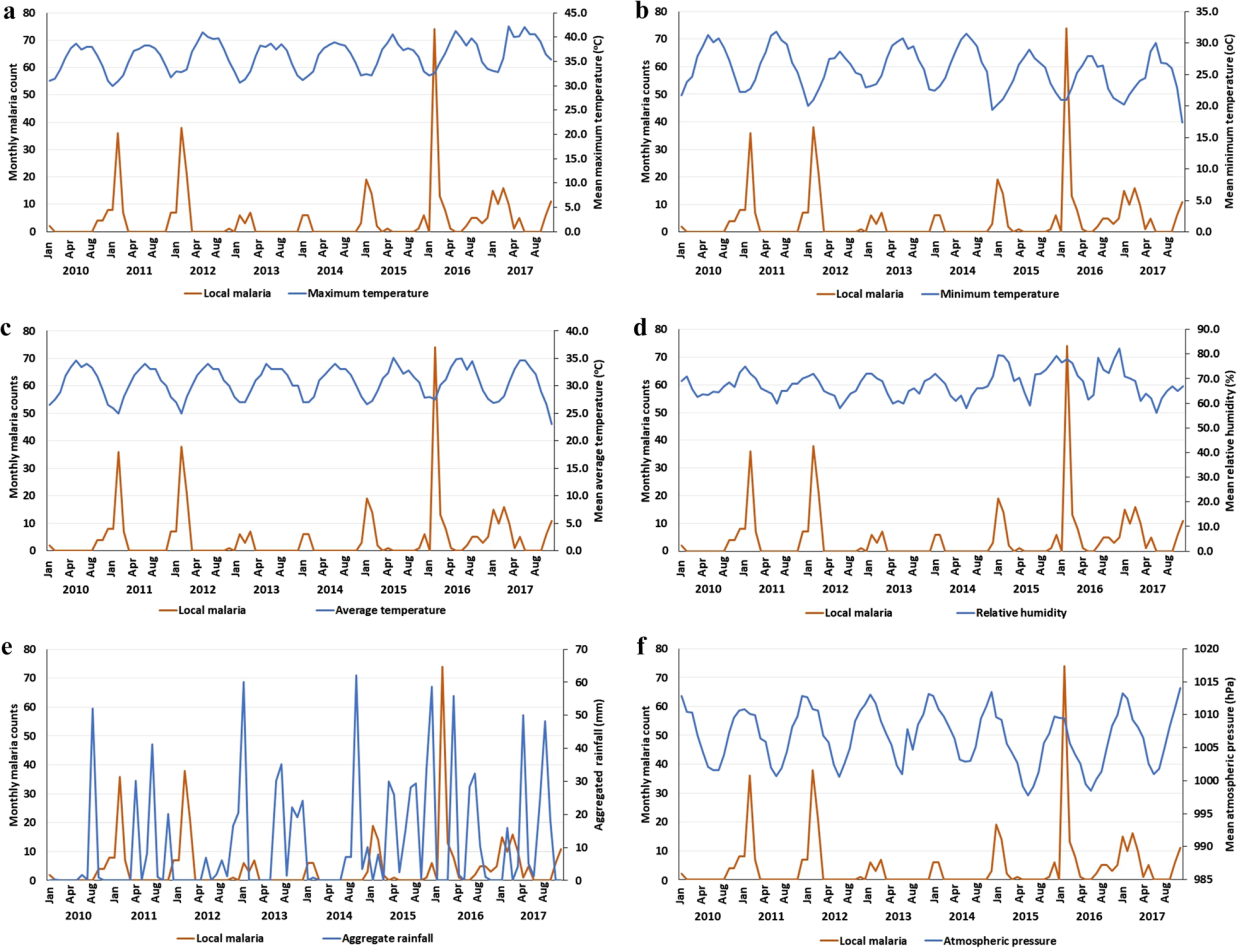
(**A**–**F**) Correlation between monthly malaria cases and climatic variables in Jazan region between 2010–2017. **A** Maximum temperature, **B** minimum temperature, **C** average temperature, **D** relative humidity, **E** aggregate rainfall, **F** atmospheric pressure

**Fig. 5:**
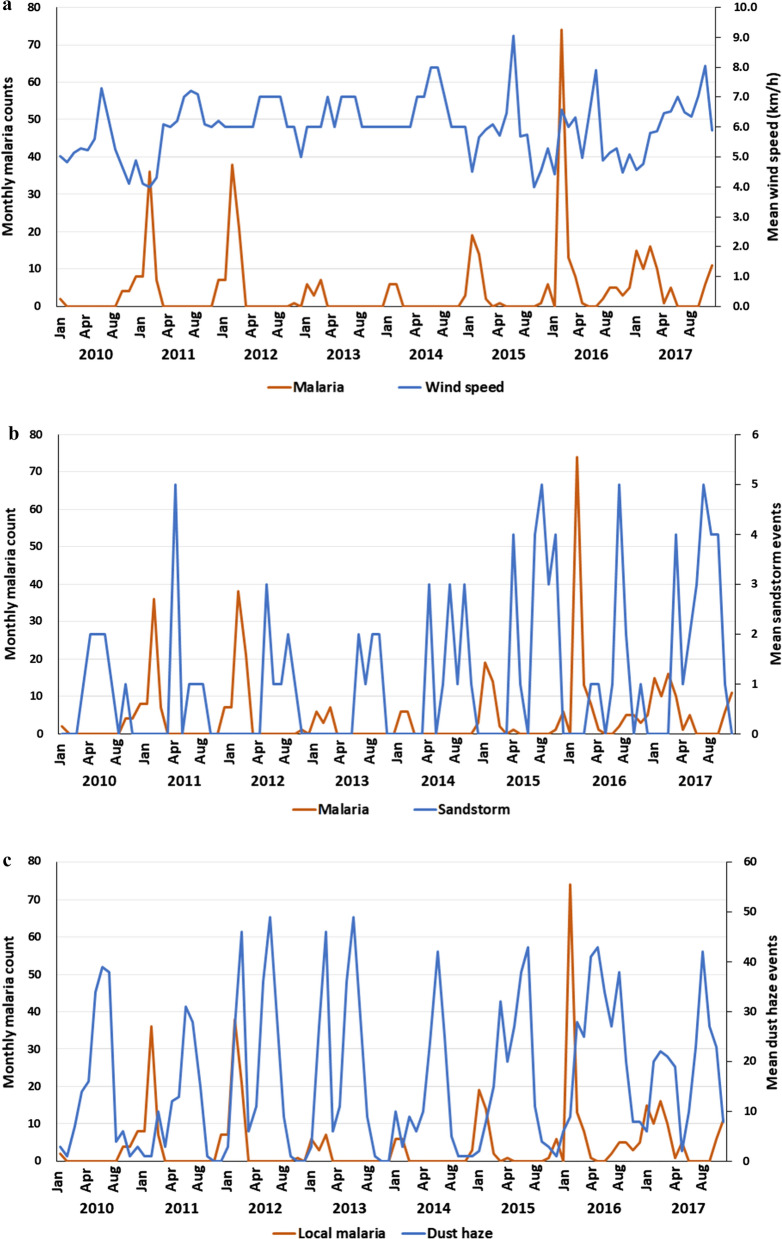
**A**–**C** Correlation between monthly malaria cases and climatic variables in Jazan region between 2010–2017. **A **Wind speed, **B** no. of sandstorms, **C** no. of dust haze events

Overall, a total of 8,718 malaria cases were reported in Jazan region between 2010 and 2017. Of these, 95.3% were imported cases and 4.7% were autochthonous (locally acquired) cases. Table 2 shows the mean monthly climatic factors for Jazan region for the period 2010–2017. Figure 4A–F and Additional file 1: Table S1 display the correlation between unlagged mean or aggregate monthly climatic variables (temperature, rainfall, relative humidity, and atmospheric pressure) and monthly malaria cases over the period 2010 to 2017. The results show that there is a significantly negative correlation of monthly malaria cases with the three temperature indices (*P* < 0.001). On the other hand, the correlation was significantly positive with mean relative humidity (r = 0.420; *P* < 0.001) and atmospheric pressure (r = − 0.281; *P* < 0.001). Similarly, Table 3 and Fig. 5A–C show a significantly negative correlation of malaria cases with number of sandstorm events (r = − 0.237; *P* < 0.01). On the other hand, the correlation of malaria cases with wind speed (r = − 0.171), aggregate rainfall (r = − 0.157) and number of dust haze events (r = -0.070) was not significant (*P* > 0.05).

**Table 2:** Mean monthly climatic factors for Jazan region for the period 2010–2017

Variables	Mean ± SD	Range
Maximum temperature (°C)	36.2 ± 3.1	30.0–42.2
Minimum temperature (°C)	25.7 ± 3.3	17.4–31.8
Average temperature (°C)	30.7 ± 2.8	23.0–35.1
Relative humidity (%)	67.8 ± 5.5	56.0–82.3
Rainfall (mm)	10.8 ± 16.6	0.0–62.1
Atmospheric pressure (hPa)	1006.3 ± 5.3	974.4–1014.1
Wind speed (km/h)	6.0 ± 1.0	4.0–9.0
Number of sandstorms	1.0 ± 1.5	0–5
Number of dust haze events	16.3 ± 14.6	0–49

**Table 3:** Pearson’s correlation coefficients of lagged climatic variables and autochthonous malaria cases reported in Jazan region between 2010 and 2017

	Maximum temperature	Minimum temperature	Average temperature	Relative humidity	Aggregate rainfall	Atmospheric pressure	Wind speed	No. of sandstorm events	No. of dust haze events
Unlagged	− 0.387^**^	− 0.477^**^	− 0.492^**^	0.420^**^	− 0.157	0.281^**^	− 0.171	− 0.257^*^	− 0.070
1-month lag	− 0.403^**^	− 0.492^**^	− 0.458^**^	0.422^**^	− 0.138	0.320^**^	− 0.318^**^	− 0.224^*^	− 0.213^*^
2-month lag	− 0.331^**^	− 0.361^**^	− 0.339^**^	0.377^**^	0.167	0.305^**^	− 0.227^*^	− 0.164	− 0.297^**^
3-month lag	− 0.090	− 0.162	− 0.071	0.238^*^	0.196	0.151	− 0.270^**^	0.115	− 0.256^*^
4-month lag	0.126	0.072	0.142	0.130	0.017	− 0.014	− 0.246^*^	0.184	− 0.162

^**^Significant correlation at *P* < 0.01

^*^Significant correlation at *P* < 0.05

Correlation of malaria cases with lagged climatic variables was assessed (Table 3). The significant correlation between lagged temperature indices and monthly malaria cases occurred at 1- and 2-month lags with a peak at 1-month lag period. Similarly, the significant correlation of humidity and wind speed with malaria cases continued to 3-month and 4-month lag periods, respectively. Moreover, other variables correlated significantly with malaria cases at specific lag periods (Table 3). However, the correlation between malaria cases and monthly aggregate rainfall was not significant at all lag periods (*P* > 0.05).

The results of ZINB regression showed both the count model coefficients and the inflation model that contained logit coefficients for predicting excess zeroes (Table 4). In Model A, the negative binomial results showed that mean minimum temperature at 1-month lag and unlagged average temperature had a significant effect on the number of autochthonous malaria cases in Jazan region. In another model (Model B) that included relative humidity instead of minimum temperature, the results showed that relative humidity was the only significant variable. When other variables (at specific lag periods) that showed significant correlation with monthly malaria cases were included instead of the related lags in a stepwise manner, different results were obtained. With regards to ZINB logit coefficients, monthly mean maximum and minimum temperature, both at 1-month lag, and unlagged average temperature were the significant determinants of monthly malaria cases identified by Model A while relative humidity and maximum temperature, both at 1-month lag, and unlagged average temperature were the significant variables in Model B. A reduced model (Model C) showed that average temperature and relative humidity were the significant determinants of malaria cases (Table 4).

**Table 4:** Results of zero-inflated negative binomial regression for the relationship between climatic variables and autochthonous malaria reported in Jazan region between 2010 and 2017

Variables	Count model (negative binomial coefficients)					Zero-inflated model (logit coefficients)				
	β	SE	95% CI	*P*	% change^†^	β	SE	95% CI	*P*	OR^‡^
Model A										
Intercept	16.593	3.809	9.023, 24.163	< 0.001^*^	–	− 21.481	8.775	− 38.919, − 4.043	0.014	–
Maximum temperature (lag 1)	− 0.095	0.099	− 0.292, 0.102	0.336	− 9.06	− 0.736	0.351	− 1.434, − 0.038	0.036^*^	0.48
Minimum temperature (lag 1)	− 0.243	0.073	− 0.388, − 0.098	0.001^*^	− 21.57	0.704	0.258	0.191, 1.217	0.006^*^	2.02
Average temperature (lag 0)	− 0.157	0.078	− 0.312, − 0.002	0.045^*^	− 14.53	0.853	0.351	0.155, 1.551	0.015^*^	2.35
Atmospheric pressure (lag 1)	− 0.131	0.182	− 0.493, 0.231	0.130	− 12.28	0.196	0.638	− 1.072, 1.464	0.758	1.22
Wind speed (lag 1)	− 0.145	0.141	− 0.425, 0.135	0.301	− 11.50	0.651	0.483	− 0.309, 1.611	0.177	1.92
No. of sandstorms (lag 0)	− 0.051	0.140	− 0.329, 0.227	0.714	− 4.97	− 0.366	0.351	− 1.064, 0.332	0.298	0.69
No. of dust haze events (lag 2)	0.001	0.127	− 0.251, 0.253	0.999	0.10	0.308	0.322	− 0.332, 0.948	0.339	1.36
Model B										
Intercept	1.683	3.260	− 4.795, 8.162	0.817	−	35.394	13.098	9.364, 61.424	0.078	−
Maximum temperature (lag 1)	− 0.016	0.135	− 0.284, 0.252	0.907	− 1.56	− 0.603	0.273	− 1.146, − 0.061	0.027^*^	0.55
Average temperature (lag 0)	− 0.152	0.087	− 0.325, 0.021	0.079	− 14.10	0.796	0.346	0.108, 1.484	0.021^*^	2.22
Relative humidity (lag 1)	0.097	0.047	− 0.004, 0.190	0.047^*^	10.19	− 0.393	0.145	− 0.681, − 0.105	0.007^*^	0.68
Atmospheric pressure (lag 1)	0.246	0.474	− 0.696, 1.188	0.603	27.94	− 1.830	1.349	− 4.511, 0.851	0.175	0.16
Wind speed (lag 1)	− 0.053	0.160	− 0.371, 0.265	0.742	− 5.16	0.261	0.518	− 0.768, 1.290	0.615	1.30
Number of sandstorms (lag 0)	− 0.017	0.169	− 0.353, 0.319	0.920	− 1.69	− 0.484	0.428	− 1.335, 0.367	0.258	0.62
Number of dust haze events (lag 2)	− 0.079	0.158	− 0.393, 0.235	0.617	− 7.60	0.530	0.423	− 0.311, 1.371	0.211	1.70
Model C										
Intercept	12.388	1.726	8.960, 15.816	< 0.001^*^	−	− 14.350	4.500	− 23.293, − 5.407	0.001	–
Maximum temperature (lag 1)	− 0.090	0.066	− 0.221, 0.041	0.171	− 8.61	− 0.090	0.066	− 0.221, 0.041	0.104	0.91
Average temperature (lag 0)	− 0.194	0.072	− 0.337, − 0.051	0.007^*^	− 17.63	0.631	0.212	0.210, 1.052	0.003^*^	1.88
Relative humidity (lag 1)	0.059	0.039	− 0.018, 0.136	0.134	6.08	− 0.347	0.173	− 0.691, − 0.003	0.045^*^	0.71

β: Regression coefficient; SE: Standard error of β; CI: Confidence interval for β

^†^ Computed as (e^β^ − 1)*100

^‡^ Computed as (e^β^)

^*^ Significant association (*P* < 0.05)

Based on AIC, Model C (AIC = 327.260) was best suited to predict the monthly autochthonous malaria cases in Jazan region compared to Model A (AIC = 330.893) and B (AIC = 337.659) and other models. Thus, ZINB logit coefficients of Model C were the final results of this regression model (Table 4). The results showed that the odds that monthly autochthonous malaria count would be zero in Jazan region increased (i.e., it is less likely that there will be malaria cases) with an increase of one °C in monthly unlagged average temperature (OR = 1.88). On the other hand, the odds that monthly malaria count would be zero decreased (i.e. it is more likely that there will be malaria cases) with an increase of 1% in relative humidity (OR = 0.71).

## Discussion

The current study revealed that malaria remains a public health problem in Jazan region, with a total of 1124 confirmed cases were reported among the febrile patients presented at healthcare centres during the study period. Since the introduction of a malaria elimination strategy in 2004, the burden of malaria in Saudi Arabia has been markedly reduced, and the country has successfully decreased the burden and geographic extent of malaria nationwide [29]. However, a limited number of malaria foci remain in Jazan and Aseer regions in southwestern Saudi Arabia [9, 30].

In the current study, the percentage of febrile subjects found positive for malaria parasites was higher than that reported by previous studies conducted in Jazan and some other regions of the country, including the Makkah region and in the Al-Ahsa governorate in Eastern province [30–33]. El Hassan et al. [9] showed a dual trend of malaria cases in Jazan between 2000 and 2014, i.e., a significant reduction in autochthonous malaria cases (from 35.3 per 10,000 population in 2000 to the lowest rate of 0.11 cases per 10,000 population in 2014) and a constant number of imported malaria cases. A similar situation was also reported in the neighboring Aseer region [30]. However, since 2015, a steady rise in malaria cases in both Jazan and Aseer regions has been noted [34, 35], and this observation is supported by the findings of the current study. Compared to the very low proportion of autochthonous cases reported annually as compared to imported cases since 2014, the current study found that 4.5% (51/1124) of the cases can be considered autochthonous, with autochthonous cases reported during the outbreak in Baysh governorate were excluded. Hence, generally, it can be said that Saudi Arabia continues to make good progress toward achieving the WHO E-2020 goal [1].

Moreover, the current study revealed that malaria transmission is still active in Jazan region with malaria cases identified in 14 governorates (out of 17) during the study period. The findings also showed that malaria in Jazan region occurs throughout the year without any obvious seasonal patterns, a finding that coincides with that reported by a previous study conducted in Aseer region [30]. Likewise, the findings also demonstrated that the monthly number of malaria cases was consistent in all governorates, except Baysh. Indeed, an outbreak of falciparum malaria was reported in Baysh governorate from November 2018 through January 2019, with an approximately two- to three-fold increase in the number of confirmed cases relative to the mean number of cases for the same months in the five preceding years plus two times the standard deviation [36, 37]. Despite a lack of authoritative information on its spread and determinants, this outbreak could be attributed to a combination of factors, including unusual heavy rainfall and flooding in Jazan region including Wadi Baysh (the largest perennial stream in Saudi Arabia) for 2–3 months before the onset of outbreak, which may have caused an increase in vector breeding sites as well as the arrival of new efficient vectors [18, 21]. Moreover, the Baysh dam, built in 2009, is one of the largest dams in the country and is situated along the mainstream of Wadi Baysh [38]. Hence, it seems that, unless there are appropriate measures in place, the construction of dams for irrigation and hydroelectric generation could intensify malaria transmission by providing breeding habitats for prominent malaria vector species, especially in areas of unstable or limited transmission [18, 39]. In addition, increased cross-border importation of malaria [34] and poor awareness of malaria prevention measures among the general population [40], as well as the emerging resistance of the parasites to treatment and the resistance of the vectors to insecticides might also have contributed to this outbreak [19]. Therefore, this malaria outbreak indicates that there is a need for rigorous impact assessments of metrological and climate change-related variables as well as dam-related environmental factors in Jazan region.

The current findings also showed that *P. falciparum* was the predominant cause of malaria in Jazan which is consistent with previous reports [35, 41]. The majority of vivax malaria cases identified by the current study were among non-Saudi patients, particularly Pakistani patients followed by Indian and Yemeni patients, as compared with only three cases among the Saudi patient group. Although *P. falciparum* is the predominant species in the neighbouring endemic country, Yemen [42, 43], the incidence of vivax malaria has been rising since 2015 [4]. In addition, it was recently estimated that over three quarters (79·5%) of the global burden of vivax malaria in 2017 was attributable to India, Pakistan and Ethiopia [4].

As regards the distribution of cases according to demographic factors, the current findings showed that malaria was present among all age groups including children below 5 years. However, the percentage of positive for malaria was the highest among patients aged 18–30 years, which corresponds with previous reports [30, 32]. This finding could be explained by the higher mobility and occupation-related factors among adult individuals, which may lead to higher exposure to mosquito bites. Similarly, the higher percentage of cases reported in the current study among males could be attributed to behavioral differences and the customs and the traditions in the country. For instance, the female population wears clothes that cover the entire body, whereas the male population tends to wear lighter clothing that exposes arms and legs thereby increasing susceptibility to mosquito bites. However, it should be borne in mind that the reported difference could be attributed to the low number of female participants involved in this study. A greater likelihood of infection among the male population has been reported by previous studies undertaken in Jazan region and elsewhere [35, 44, 45]. In contrast, other studies have demonstrated that women are 40% more likely than men to contract malaria and that pregnant women are at greater risk of malaria infection and also suffer higher morbidity and mortality [46, 47].

In addition, the findings showed significantly higher percentage of malaria among non-Saudi patients as compared with Saudi patients, with more than half (54.2%) of the cases found among Yemeni patients. These findings are consistent with those previously reported for Jazan [9, 35]. Moreover, among a total of 318 malaria cases reported in Makkah region between 2008 and 2011, non-Saudi patients accounted for 95%, with Pakistanis, Nigerians, and Indians accounting for 62.0% [32]. Similarly, out of 3151 malaria cases reported in all regions of the country in 2017, 2974 (95%) were imported cases, while the remaining 177 cases were autochthonous [3].

The porous international borders between countries represent a major challenge to the elimination of malaria, with the Saudi Arabia border with Yemen being a typical example [34, 48]. The migration of malaria-infected humans has been shown to rapidly undermine gains made in malaria control efforts [49]. In Saudi Arabia, imported malaria remains a major problem with a constant flow of imported malaria, mostly among immigrant workers from south Asia (Pakistan, India and Bangladesh), East Africa (Sudan and Ethiopia), and Yemen [30, 50]. In an effort to address this problem, a collaborative Saudi Arabia and Yemen cross-border joint-programme of malaria control was launched in 2002 and remained operational until 2014 before it ceased due to the armed crisis in Yemen. As a result, the incidence of malaria in Jazan region dramatically decreased until 2014. However, between 2015 and 2017, it has been estimated that 32% of all imported infections detected in Saudi Arabia were of Yemeni origin [34]. Coincidentally, the incidence of the locally acquired cases in Saudi Arabia increased [9, 35]. Moreover, it should be noted that the majority of non-Saudi immigrant workers in the region reside in areas with poor housing and environmental settings that may favor mosquito breeding and malaria transmission. In general, malaria has been traditionally considered as less of a problem in urban areas compared with peri-urban slums**/**settlements and rural areas [51].

The association between climatic factors and malaria transmission has been extensively studied and the results are quite varied, thereby indicating that this association is complex. Nonetheless, such information from Saudi Arabia and Jazan region is lacking. Although rainfall is considered a critical factor that provides suitable breeding sites for mosquitoes, temperature is a key driver of many vital traits of the development and the life cycle of both the parasite and the mosquito [52–54]. The current study showed that average temperature and relative humidity were the significant climatic determinants of autochthonous malaria in Jazan region. The findings on temperature are consistent with those in previous studies that demonstrated that temperature below 27 °C was associated with a high incidence rate, while a temperature over 30 °C was related to the lowest incidence rates [55, 56]. The optimal temperature for malaria transmission ranges between 16 °C and 34 °C and peak transmission occurs at 25 °C [53], which is 5–7 °C cooler than that estimated by other previous studies [57, 58]. Likewise, a positive relationship between malaria and relative humidity has been demonstrated by previous studies elsewhere [54, 55, 59]. High humidity (> 60%) is essential to enhance the lifespan of the mosquito and the development of the parasite in mosquitoes [60].

The current study has some limitations that should be considered when interpreting the above findings. First, data on the distribution of malaria cases between April 2018 and January 2019 came from passive case detection among febrile patients presenting for healthcare at participating health facilities and this limited the ability to detect malaria infection among parasitaemic but asymptomatic individuals. It should be mentioned that according to the Saudi Ministry of Health’s statistics, 184,734 individuals were examined for malaria in Jazan region during 2018, and 1516 of them were found positive with a positivity rate of 0.82% [36]. As a result, the current findings should be interpreted with caution and evaluated as part of the larger context of the malaria situation in the region. Second, as malaria in Jazan is mostly imported, the association between climatic variables and malaria had to rely on small monthly number of autochthonous malaria reported during the period of 2010–2017. Third, the positive cases reported in this study involved only eight out of 17 governorates of Jazan region. Indeed, Farasan Island and Fayfa highlands were reported malaria-free [18], while all other governorates share similar epidemiological characteristics. Thus, the findings can be generalized to include those governorates; however, further studies are required to investigate this conjecture.

## Conclusions

This study revealed that Saudi Arabia still has a long way to go to eliminate malaria from the entire country. Despite the continued intensive efforts being made by the Saudi health authorities to combat malaria, numbers of malaria cases are still being reported in most governorates of Jazan region. However, the identification and monitoring of malaria transmission foci would enable control efforts to be intensified and focused on specific areas, and therefore might also ultimately lead to the elimination of residual malaria in the whole region.

Promotion of operational research is crucial to identify and determine the feasibility of innovative and integrated measures that would maximize the effectiveness of control strategies against cross-border malaria as well as to generate and make best use of data to guide malaria elimination efforts. Moreover, further studies on modelling the relationship of malaria and its vectors with metrological and climate change-related factors in Jazan region are essential to predict malaria outbreaks and to understand the transmission dynamics of the disease in southwestern Saudi Arabia.

## Supplementary Information

**Additional file 1: Table S1.** Pearson’s correlation coefficient matrix of unlagged monthly climatic variables and autochthonous malaria cases reported in Jazan region between 2010 and 2017

## Data Availability

The data that support the findings of this study are available from the corresponding author upon reasonable request.
