# Supplementary material for: Residual malaria in Jazan region, southwestern Saudi Arabia: the situation, challenges and climatic drivers of autochthonous malaria
Source: Malar J. 2021 Jul 13;20:315. doi: 10.1186/s12936-021-03846-4 (PMC8276496; doi:10.1186/s12936-021-03846-4)
Supplement: Supplementary file 1 — Additional file 1: Table S1. Pearson’s correlation coefficient matrix of unlagged monthly climatic variables and autochthonous malaria cases reported in Jazan region between 2010 and 2017 [file 12936_2021_3846_MOESM1_ESM.docx]

**Table S1** **Pearson’s correlation coefficient matrix of unlagged climatic variables and autochthonous** **malaria cases reported in Jazan region between 2010–2017.**

|  | Malaria cases | Maximum temperature | Minimum temperature | Average temperature | Relative humidity | Aggregate rainfall | Atmospheric pressure | Wind speed | No. of sandstorm events | No. of dust haze events |
| --- | --- | --- | --- | --- | --- | --- | --- | --- | --- | --- |
| Malaria cases | 1 |  |  |  |  |  |  |  |  |  |
| Maximum temperature | -0.387^**^ | 1 |  |  |  |  |  |  |  |  |
| Minimum temperature | -0.477^**^ | 0.706^**^ | 1 |  |  |  |  |  |  |  |
| Average temperature | -0.492^**^ | 0.862^**^ | 0.868^**^ | 1 |  |  |  |  |  |  |
| Relative humidity | 0.420^**^ | -0.653^**^ | -0.731^**^ | -0.616^**^ | 1 |  |  |  |  |  |
| Aggregate rainfall | -0.157 | 0.148 | 0.085 | 0.175 | 0.079 | 1 |  |  |  |  |
| Atmospheric pressure | 0.281^**^ | -0.664^**^ | -0.672^**^ | -0.743^**^ | 0.406^**^ | -0.186 | 1 |  |  |  |
| Wind speed | -0.171 | 0.507^**^ | 0.519^**^ | 0.463^**^ | -0.633^**^ | 0.080 | -0.416^**^ | 1 |  |  |
| No. of sandstorm events | -0.237^*^ | 0.566^**^ | 0.372^**^ | 0.459^**^ | -0.188 | 0.343^**^ | -0.355^**^ | 0.121 | 1 |  |
| No. of dust haze events | -0.070 | 0.505^**^ | 0.416^**^ | 0.468^**^ | -0.347^**^ | 0.038 | -0.550^**^ | 0.452^**^ | 0.234^*^ | 1 |

^**^ Significant correlation at *P* < 0.01

^*^ Significant correlation at *P* < 0.05
